# Two-Stage Multi-Frame Fusion Model for Radar Anti-Jamming Target Detection with Range-Doppler Maps

**DOI:** 10.3390/s26206419

**Published:** 2026-10-11

**Authors:** Xiaoying Chen, Ying Liu

**Affiliations:** University of Chinese Academy of Sciences, Beijing 100049, China; chenxiaoying21@mails.ucas.ac.cn

**Keywords:** active deception jamming, anti-jamming detection, cognitive radar, range-Doppler maps, spatiotemporal features

## Abstract

Mainlobe active deception jamming significantly degrades radar target detection in complex electromagnetic environments. Current anti-jamming detection approaches inadequately exploit the spatiotemporal information inherent in target echoes, thereby fundamentally limiting their capacity for reliable detection and robust true–false discrimination. Therefore, this paper proposes a trajectory-guided two-stage multi-frame fusion detection (TSMF-Det) model based on range-Doppler (R-D) maps. Initially, a lightweight candidate detector is utilized to generate high-recall candidate boxes, which are then linked into cross-frame trajectories via temporal association. Subsequently, a spatiotemporal refinement network (STRNet) is designed to aggregate multi-frame temporal and multi-scale spatial features, enabling fine-grained true–false target discrimination and bounding-box refinement. Finally, an adaptive peak-center fusion module dynamically achieves high-precision estimation of range and velocity parameters. Experiments on a comprehensive dataset encompassing six typical active deception jamming scenarios demonstrate that TSMF-Det yields an average detection probability of 0.97. Comparative experiments show that TSMF-Det maintains stable target detection under strong jamming while providing effective false-target rejection and accurate range–velocity estimation. By exploiting multi-frame spatiotemporal features, TSMF-Det provides an effective anti-jamming detection framework for cognitive radar systems.

## 1. Introduction

Modern radar systems increasingly operate amid dense electromagnetic activity. In particular, mainlobe active deception jamming introduces two primary difficulties for target detection: weak target echoes under a reduced signal-to-jamming-plus-noise ratio (SJNR) and resource-consuming false responses [1]. Because mainlobe jamming occupies the same angular sector as true targets, traditional suppression methods based on spatial filtering inevitably attenuate true target echoes. Furthermore, within the anti-jamming framework of cognitive radar, closed-loop waveform design and resource scheduling require complete state-space information of the electromagnetic confrontation [2]. If aggressive suppression is used in preprocessing to completely remove jamming, the back-end decision-making system will lose critical prior knowledge about the adversary. Therefore, developing an end-to-end multi-target detection paradigm that preserves jamming distribution information, precisely distinguishes true from false targets, and extracts their motion parameters is crucial for next-generation cognitive radar systems.

Conventional anti-deception methods generally follow a serial procedure consisting of jamming analysis, suppression or discrimination, and subsequent target detection. For interrupted-sampling repeater jamming (ISRJ), time-frequency differences between target echoes and retransmitted segments have been exploited to design bandpass filters for false-target suppression [3]. Complementary signal sets have also been employed for waveform-domain moving-target detection and multi-ISRJ suppression [4]. Frequency-agile waveforms and random phase coding have also been combined with sparse recovery to increase the structural separability of target and jamming components under high-duty-cycle interference [5,6]. In spatially distributed systems, multistatic diversity has been used to reduce the coherent integration advantage of deceptive signals [7], while full-polarization radar networks have employed multi-channel scattering features for cooperative true–false target discrimination [8]. Motion-related amplitude evolution during long-time coherent integration has further been investigated using the Radon–Fourier transform [9]. Despite their physical interpretability, the effectiveness of these approaches often depends on accurate jamming models, specialized waveform or hardware configurations, and reliable intermediate processing. Their generalization therefore degrades when jamming parameters vary, multiple responses overlap densely, or errors propagate through the serial processing stages.

Deep learning provides a data-driven alternative by jointly learning feature extraction and decision functions. Early studies processed one-dimensional raw echoes with convolutional neural networks to perform end-to-end anti-jamming detection [10], and were subsequently extended to mixed-source signal separation, target localization, and ISRJ parameter estimation [11,12,13]. Time-frequency spectrograms have also been integrated with object detectors for the localization and parameter estimation of multiple deception-jamming types [14,15,16]. Multi-domain feature fusion and Wasserstein generative adversarial networks were introduced to improve robustness under limited training data [17,18]. At the range-profile level, PMPE-Net incorporated multimodal perception, feature decoupling, and differentiable pulse compression into an end-to-end architecture [19]. Despite these advances, most methods remain limited to single-pulse representations. Such inputs inherently lack Doppler information or suffer from local texture degradation under low SJNR conditions, restricting complete range–velocity estimation and reliable separation of densely distributed responses.

The R-D domain provides a more direct representation of target range, velocity, and coherent energy distribution. Existing studies have combined moving-target detection with convolutional detection for ISRJ mitigation [20], and have applied improved YOLOX-based models to target detection from R-D maps [21]. Multi-scale feature extraction and attention mechanisms have further enhanced weak-target representation and localization under barrage jamming or clutter [22,23]. Although these methods exploit the two-dimensional structure of R-D data, most rely on isolated frames. True targets and high-fidelity false targets often exhibit highly similar local morphologies. In such cases, single-frame spatial cues fail to provide stable discrimination, particularly during transient energy fluctuations, noise masking, and strong jamming.

Multi-frame observations offer an additional source of evidence through temporal continuity and trajectory evolution. Track-before-detect methods have exploited sequential weak measurements for joint target detection and state estimation [24]. Spatiotemporal networks constructed from consecutive millimeter-wave radar range-azimuth heatmaps have demonstrated the value of combining three-dimensional convolutions with Transformers [25]. ConvLSTM-based detectors have been used to capture temporal dependencies and improve small-target detection [26], while RECORD employs recurrent memory for online detection from successive radar frames [27]. For low-radar-cross-section target detection, consecutive R-D trajectories have been processed by three-dimensional deep convolutional networks together with low-threshold CFAR to suppress sidelobes and clutter-induced false alarms [28,29]. Cascaded frameworks using CFAR candidate screening, deep refinement, and multi-frame association have also improved detection stability in strong clutter [30]. For deception-jamming discrimination, multi-pulse frequency-response features and multi-CPI joint decisions have been used for false-target recognition [31], while multi-CPI frequency-response reconstruction errors have been used to characterize temporal variations in target scattering centers [32]. These studies confirm that temporal context can reduce the instability of frame-wise detection. However, within the field of radar target detection against deception jamming, there remains a distinct lack of research that effectively leverages multi-frame spatiotemporal information to achieve precise true–false discrimination and robust localization.

Radar anti-jamming detection in complex electromagnetic environments faces the key challenge of effectively exploiting the spatiotemporal information contained in multi-frame echoes. To address this challenge, this study operates in the range-Doppler (R-D) domain, where the physical responses of targets and jamming are jointly represented. R-D matrices with different dimensions are normalized and converted into a unified image representation for subsequent network processing. On this basis, a trajectory-guided two-stage multi-frame fusion detection framework, termed TSMF-Det, is developed. It first combines a lightweight detector with temporal association to construct high-recall candidate trajectories, and then employs a spatiotemporal refinement network (STRNet) to aggregate multi-frame temporal and multi-scale spatial features for robust target detection and true–false discrimination. This work makes the following primary contributions:

1. Two-Stage Multi-Frame Detection Architecture:A cascaded framework is designed to couple high-recall candidate generation with spatiotemporal refinement. By exploiting cross-frame associations together with multi-frame temporal and multi-scale spatial features, the framework improves true–false target discrimination and detection robustness.
2. Adaptive Physical Parameter Extraction: An adaptive peak-center fusion module is designed to address the accuracy limitations of single-prior parameter extraction strategies. By dynamically fusing bounding-box centers and local amplitude peaks, the module ensures precise range and velocity estimation.
3. Comprehensive Experimental Validation: A multi-frame R-D image dataset covering six typical active deception jamming scenarios is constructed. Comparative and ablation experiments demonstrate the advantages of TSMF-Det in detection robustness, false-target rejection, and physical parameter estimation accuracy.

## 2. Materials

### 2.1. Radar and Jamming Signal Models

Based on a typical pulse-Doppler radar architecture, this section formulates the mathematical models for the transmitted linear frequency modulation (LFM) signal, target echoes, and six representative types of active deception jamming. The transmitted LFM pulse is modeled as:

(1) $$s_{T}\left(t\right)=A_{T}\mathrm{rect}\left(\frac{t}{T_{p}}\right)\exp\left\{j2\pi \left(f_{c}t+\frac{K}{2}t^{2}\right)\right\}$$

where rect(·) is a rectangular window, while $A_{T}$, $f_{c}$, $T_{p}$, and *B* represent the amplitude, carrier frequency, pulse width, and signal bandwidth, respectively, with $K=B/T_{p}$ representing the chirp rate.

For a target located at range *R* with velocity *v*, the time delay τ=2R/c and Doppler frequency $f_{d}=2v/\lambda$ are determined by the speed of light *c* and radar wavelength $\lambda =c/f_{c}$. Under active deception jamming, the received echo of a single pulse is formulated as:

(2) $$s_{R}\left(t,q\right)=A_{r}s_{T}\left(t-\tau \right)\exp\left(j2\pi f_{d}qT_{r}\right)+\sum_{r=1}^{N_{J}}j_{r}\left(t,q\right)+n\left(t,q\right)$$

where *q* denotes the slow-time pulse index, $T_{r}$ is the pulse repetition interval (PRI), and $A_{r}$ represents the complex amplitude of the echo. The term $j_{r}\left(t,q\right)$ is the *r*-th of the $N_{J}$ total active jamming components, and n(t,q) accounts for the additive receiver noise.

Modern active deception jammers commonly employ digital radio-frequency memory (DRFM) to generate false targets during search or to pull tracking gates away from the true target [33,34]. The six jamming mechanisms considered in this study are described below.

Dense false target (DFT) jamming relies on time-delay modulation, retransmitting the intercepted radar signal multiple times to generate coherent false targets in the range domain. The mathematical model is formulated as:

(3) $$j_{\mathrm{DFT}}\left(t,q\right)=A_{J}\sum_{i=1}^{M_{f}}s_{T}\left(t-\tau_{0}-\Delta \tau_{i}\right)$$

where $A_{J}$ and $M_{f}$ denote the jamming amplitude and the total count of false targets, respectively; $\tau_{0}$ defines the baseline retransmission delay, while $\Delta \tau_{i}$ is the time delay of the *i*-th false target.

Interrupted-sampling repeater (ISR) jamming periodically extracts short segments of the intercepted pulse and retransmits each segment multiple times. Let $T_{u}$ denote the sampling period, $T_{J}$ the duration of a single sampled slice, and $M_{r}$ the number of retransmissions. The ISR jamming signal is given by:

(4) $$j_{\mathrm{ISR}}\left(t,q\right)=A_{J}\sum_{m=1}^{M_{r}}\sum_{n=0}^{N_{s}-1}\mathrm{rect}\left(\frac{t-\tau_{0}-nT_{u}-mT_{J}}{T_{J}}\right)s_{T}\left(t-\tau_{0}-mT_{J}\right)$$

where $N_{s}$ denotes the total number of slices sampled per pulse, uniquely determined by the radar pulse width and the sampling interval.

Chopping-and-interleaving (CI) jamming follows a chop-reorder-retransmit process. The intercepted pulse is divided into $N_{c}$ equal segments of duration $T_{s}$, which are rearranged according to a permutation $\pi \left(k\right)\in \left\{0,1,\ldots ,N_{c}-1\right\}$. The signal model is expressed as:

(5) $$j_{\mathrm{CI}}\left(t,q\right)=A_{J}\sum_{k=0}^{N_{c}-1}s_{T}\left[t-\tau_{0}-\left(k-\pi \left(k\right)\right)T_{s}\right]\mathrm{rect}\left(\frac{t-\tau_{0}-kT_{s}-T_{s}/2}{T_{s}}\right)$$

Smeared-spectrum (SMSP) jamming synthesizes a sequence of time-compressed subpulses whose chirp rate is increased by a factor of $N_{S}$. Each subpulse has duration $T_{p}/N_{S}$, and the $N_{S}$ subpulses are concatenated to preserve the total pulse duration. The SMSP model is formulated as:

(6) $$j_{\mathrm{SMSP}}\left(t,q\right)=A_{J}\sum_{i=0}^{N_{S}-1}\mathrm{rect}\left(\frac{N_{S}\left(t-\tau_{i}\right)}{T_{p}}\right)\exp\left\{j2\pi \left[f_{c}\left(t-\tau_{i}\right)+\frac{N_{S}K}{2}{\left(t-\tau_{i}\right)}^{2}\right]\right\}$$

where $\tau_{i}=\tau_{0}+iT_{p}/N_{S}$ defines the time delay of the *i*-th subpulse.

Comb-spectrum (COMB) jamming modulates the intercepted waveform with a set of uniformly spaced frequency components, yielding a coherent multi-frequency response:

(7) $$j_{\mathrm{COMB}}\left(t,q\right)=s_{T}\left(t-\tau_{0}\right)A_{J}\sum_{i=1}^{M_{c}}\exp\left(j2\pi f_{i}t\right)$$

where $M_{c}$ denotes the number of modulating frequency components, $f_{i}=f_{0}+\left(i-1\right)\Delta f$ represents the *i*-th frequency, and Δf is the adjacent frequency interval.

Range–velocity gate pull-off (RVGPO) jamming synchronously controls retransmission delay and Doppler shift to capture and subsequently displace the radar tracking gates. Its signal model is:

(8) $$j_{\mathrm{RVGPO}}\left(t,q\right)=A_{J}s_{T}\left[t-\tau_{0}-\Delta \tau_{J}\left(q\right)\right]\exp\left[j2\pi \Delta f_{J}\left(q\right)qT_{r}+j\phi_{J}\right]$$

where $\Delta \tau_{J}\left(q\right)$ and $\Delta f_{J}\left(q\right)$ are the pulse-dependent delay and Doppler offsets imposed by the jammer, respectively, and $\phi_{J}$ is the initial phase.

### 2.2. Signal Preprocessing and Problem Formulation

The two-dimensional R-D representation jointly preserves range, velocity, and coherent-energy information. Compared with one-dimensional time-domain echoes, it provides a more informative domain for separating target and deception-jamming responses. For each coherent processing interval (CPI), the received signal is therefore processed by fast-time pulse compression (PC) followed by slow-time coherent integration.

Assume that one CPI contains *N* pulses and that each pulse contains *M* fast-time samples. The sampled data are denoted as $X=\left[x\left[m,q\right]\right]\in C^{M\times N}$, where *m* and *q* denote the fast-time and slow-time indices, respectively. Let s[m] be the sampled transmitted baseband waveform and $h\left[m\right]=s^{*}\left[-m\right]$ the matched-filter impulse response, where ${\left(\cdot \right)}^{*}$ denotes complex conjugation. The output is:

(9) $$x_{\mathrm{pc}}\left[k,q\right]=\sum_{m=0}^{M-1}x\left[m,q\right]h\left[k-m\right]$$

where *k* is the range cell index. Subsequently, a discrete Fourier transform (DFT) is performed across different pulses within the same range cell to obtain the R-D complex matrix:

(10) $$X_{\mathrm{RD}}\left[k,l\right]=\sum_{q=0}^{N-1}x_{\mathrm{pc}}\left[k,q\right]\exp\left(-j\frac{2\pi lq}{N}\right)$$

where *l* denotes the Doppler cell index. The R-D amplitude matrix is $A_{\mathrm{RD}}\left[k,l\right]=\left|X_{\mathrm{RD}}\left[k,l\right]\right|$. The discrete matrix indices (k,l) can then be linearly mapped to the physical range and velocity.

To quantitatively assess the processing gain, an SNR evolution model is established. Let ${\mathrm{SNR}}_{\mathrm{raw}}$ denote the raw echo SNR. Following pulse compression and coherent integration, the accumulated detection SNR in the R-D domain, denoted as ${\mathrm{SNR}}_{\mathrm{RD}}$, can be expressed as:

(11) $${\mathrm{SNR}}_{\mathrm{RD}}={\mathrm{SNR}}_{\mathrm{raw}}+10\log_{10}\left(BT_{p}\right)+10\log_{10}\left(N\right)$$

Thus, for a given ${\mathrm{SNR}}_{\mathrm{raw}}$, R-D domain coherent processing significantly amplifies the energy of the true target while synchronously enhancing the highly coherent features of the deception jamming. This mapping establishes an optimal observation space for executing deep feature decoupling.

To prevent the loss of detail in weak targets caused by the extreme dynamic range of $A_{\mathrm{RD}}$, an adaptive dynamic range normalization is applied to the logarithmic R-D matrix. Based on the estimation of the background noise floor, a dynamic lower bound is established to clip low-amplitude background values. The effective amplitude is linearly mapped to [0,1], quantized into an 8-bit grayscale image, and resized via bicubic interpolation. This preserves target mainlobes and jamming modulation patterns while suppressing background fluctuations.

**Figure 1:**
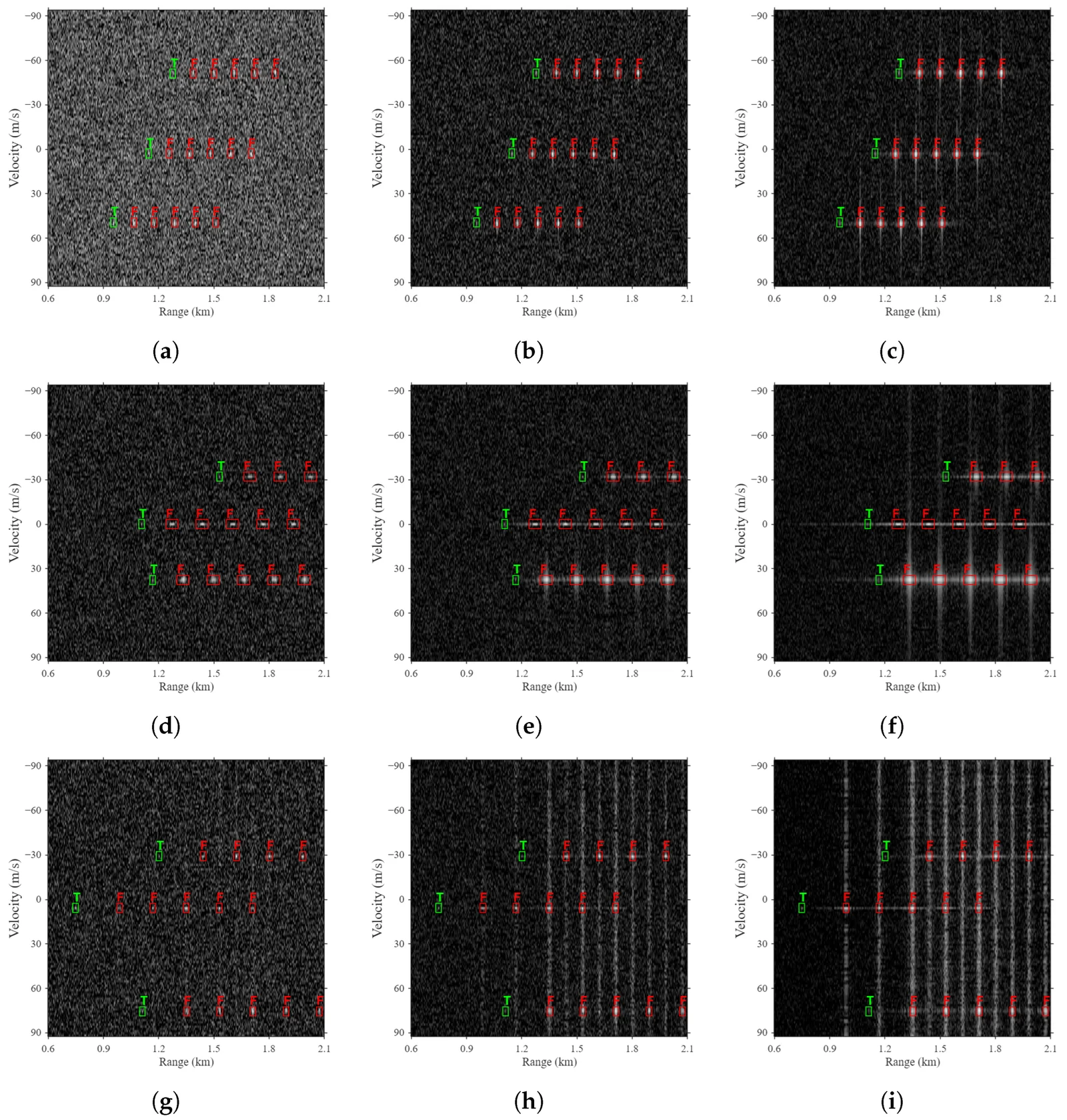
R-D domain dataset samples under various deception jamming scenarios and ${\mathrm{SNR}}_{\mathrm{RD}}$/JSR conditions. The green bounding boxes (labeled ‘T’) denote the true targets, while the red bounding boxes (labeled ‘F’) denote the deceptive false targets. (**a**–**c**), (**d**–**f**), and (**g**–**i**) correspond to DFT, ISR, and CI jamming, respectively. For each jamming type, the three columns represent varying conditions with $\left({\mathrm{SNR}}_{\mathrm{RD}},\mathrm{JSR}\right)$ set to (12,4)dB, (16,12)dB, and (20,20)dB, respectively.

**Figure 2:**
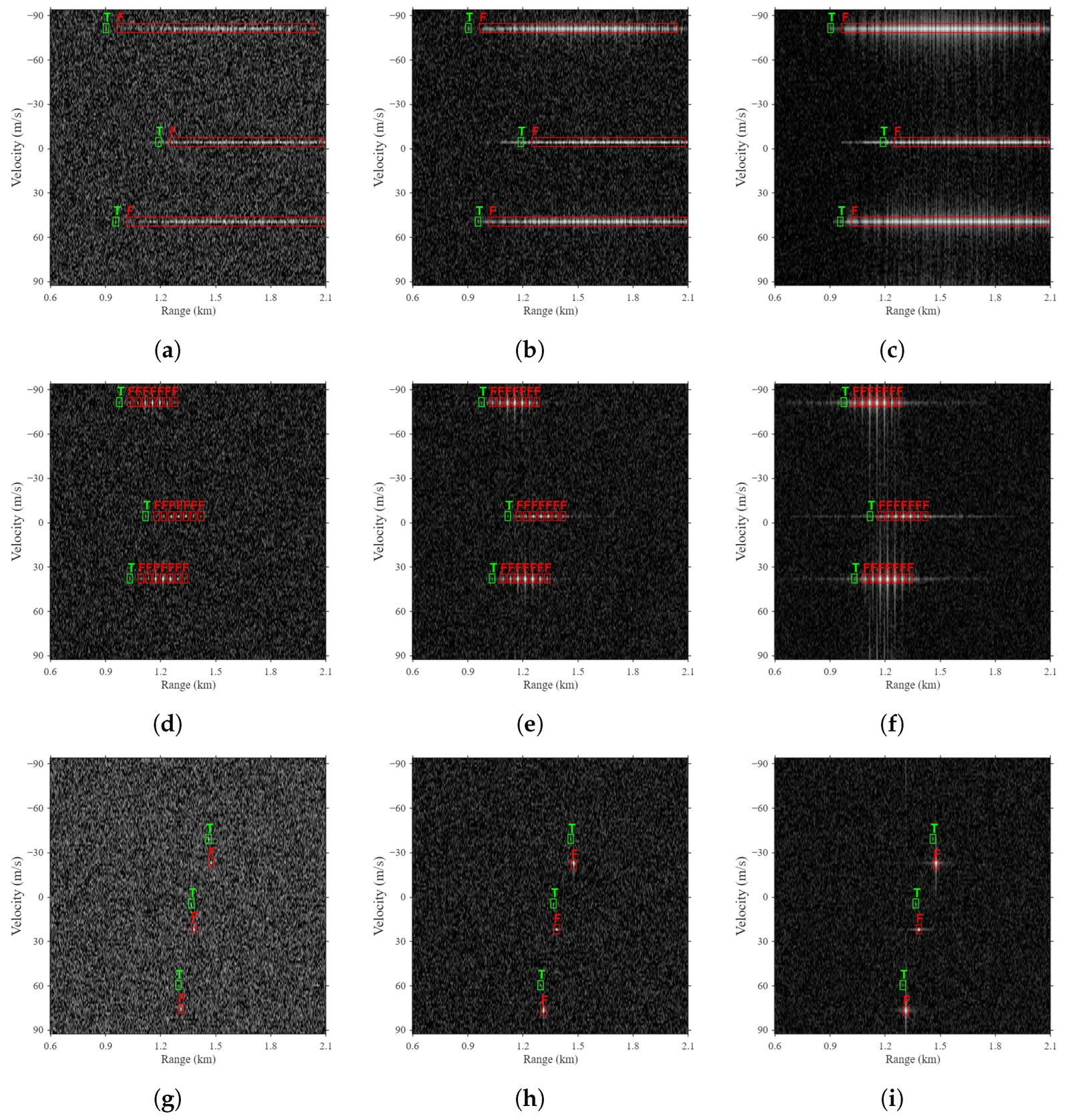
R-D domain dataset samples under additional deception jamming scenarios and ${\mathrm{SNR}}_{\mathrm{RD}}$/JSR conditions. The green bounding boxes (labeled ‘T’) denote the true targets, while the red bounding boxes (labeled ‘F’) denote the deceptive false targets. (**a**–**c**), (**d**–**f**), and (**g**–**i**) correspond to SMSP, COMB, and RVGPO jamming, respectively. For each jamming type, the three columns represent varying conditions with $\left({\mathrm{SNR}}_{\mathrm{RD}},\mathrm{JSR}\right)$ set to (12,4)dB, (16,12)dB, and (20,20)dB, respectively.

As illustrated in Figure 1 and Figure 2, the six jamming mechanisms produce distinct R-D domain responses. DFT jamming generates dense, discrete false peaks through multiple delay replicas. ISR jamming also forms dense and periodic range-domain false targets, but is accompanied by localized energy spreading. CI disrupts intrapulse phase continuity and leads to strip-like broadening in the Doppler dimension. SMSP produces extended energy smearing because of chirp-rate mismatch. COMB yields regularly spaced multi-peak responses, whereas RVGPO generates false responses that resemble true targets but evolve along displaced range and velocity trajectories. However, these spatial features degrade significantly in complex adversarial environments. Under low SNR conditions, target responses can be obscured by noise, whereas high-intensity jamming causes severe semantic masking of weak targets. Consequently, relying solely on the 2D spatial semantics of isolated single frames inevitably leads to true–false aliasing, thereby increasing missed detections and false alarms. This highlights the critical need for robust algorithms capable of discriminating targets by exploiting multi-frame spatiotemporal consistency.

## 3. Methodology

### 3.1. Overall Framework

**Figure 3:**
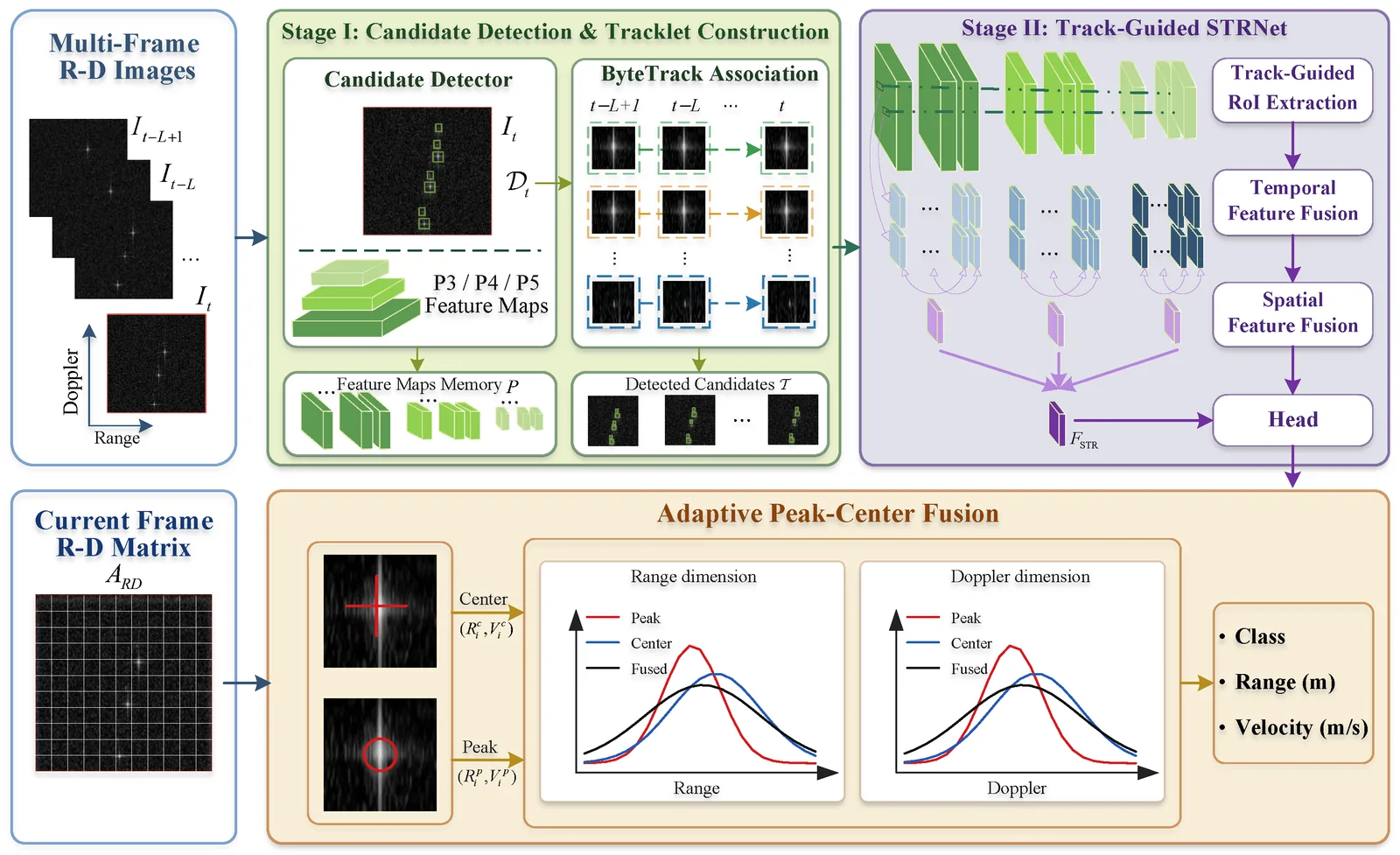
Overall architecture of the proposed trajectory-guided two-stage multi-frame detection framework. In the adaptive peak-center fusion module, the red cross denotes the geometric center of the refined bounding box, while the red circle highlights the local amplitude peak.

To achieve robust target detection and jamming environment perception under complex jamming conditions, a trajectory-guided two-stage detection framework is proposed. The overall architecture, illustrated in Figure 3, takes a sequence of R-D images $I=\{I_{t-L+1},\ldots ,I_{t}\}$ as input, where *L* denotes the temporal window length and $I_{t}$ represents the current frame. During Stage I, a candidate detector applies a low-confidence threshold to maximize true-target recall, simultaneously retaining the P3, P4, and P5 feature maps from its pyramid. A ByteTrack-based association module then links the current-frame candidates with historical observations to construct stable tracklets. In Stage II, these cross-frame trajectories guide the region-of-interest (RoI) feature extraction across multiple pyramid levels. The proposed spatiotemporal refinement network (STRNet) sequentially executes temporal feature fusion and spatial feature fusion, ultimately outputting a refined category and bounding box for the corresponding target in the current frame. Beyond robust true–false discrimination and localization, accurate estimation of the target range and velocity is also essential for radar target detection. Therefore, the framework introduces an adaptive peak-center fusion module. Based on the refined bounding box produced by STRNet, this module extracts a geometric center and a local amplitude peak specifically within the bounded region of the original current-frame R-D matrix, serving as complementary physical priors. By leveraging the trajectory-level spatiotemporal representations generated by STRNet, the module predicts dimension-specific fusion weights. The subsequent adaptive fusion of these physical priors yields accurate range and velocity estimates for true targets.

### 3.2. Stage I: Candidate Detection and Tracklet Construction

Stage I employs the efficient and lightweight YOLO26n detector [35] to extract candidate boxes from each input R-D image. Rather than producing definitive final predictions, this stage is designed to function as a high-recall region proposal mechanism. By utilizing a low confidence threshold $\tau_{\det}$, the detector retains true targets, active deception responses, and a limited number of background false alarms for subsequent multi-frame analysis. Specifically, the candidate set extracted from frame *t* after threshold filtering is defined as $D_{t}={\left\{d_{t,j}\right\}}_{j=1}^{M_{t}}$, where $M_{t}$ is the total number of retained candidates. Each candidate $d_{t,j}$ is parameterized by its bounding box $B_{t,j}$, confidence score $s_{t,j}$, and category prediction $c_{t,j}$.

Simultaneously, the detector outputs multi-scale feature maps ${\left\{P_{t}^{s}\right\}}_{s\in S}$, where S={3,4,5} denotes the pyramid levels. Shallow features retain detailed local texture and spatial information, whereas deeper features provide larger receptive fields and stronger semantic representations. These feature maps are cached for trajectory-guided RoI sampling in Stage II, thereby avoiding repeated backbone computation.

To establish spatial correspondences across consecutive frames, Stage I integrates the ByteTrack algorithm to organize isolated frame-wise candidates into continuous temporal tracklets [36]. ByteTrack is selected for its robust dual-stage data association strategy. By effectively incorporating low-score detections, it significantly alleviates trajectory fragmentation caused by target energy fluctuations or localized jamming.

Assuming that *N* active trajectories have been associated, the output tracklet for the *i*-th active trajectory (i∈{1,2,…,N}) over a temporal window of length *L* is formalized as a sequence of bounding boxes:

(12) $$T_{i}=\left\{B_{i,t-L+1},B_{i,t-L+2},\ldots ,B_{i,t}\right\}$$

Ultimately, this tracklet construction provides a cross-frame spatial index. It ensures that the localized features of the same target across continuous frames are geometrically aligned, supplying the essential spatial priors for the deep spatiotemporal feature refinement in Stage II.

### 3.3. Stage II: Track-Guided Spatiotemporal Refinement Network

STRNet performs deep spatiotemporal feature fusion to improve category discrimination and bounding-box accuracy. It comprises four components: track-guided RoI extraction, multi-frame temporal feature fusion, cross-scale spatial feature fusion, and classification and regression heads. Each component addresses a distinct aspect of multi-frame R-D detection: track-guided RoI extraction preserves cross-frame correspondence, temporal fusion mitigates transient frame-wise fluctuations, cross-scale spatial fusion integrates complementary multi-scale information, and the refinement heads convert the fused representation into category predictions and refined locations.

#### 3.3.1. Track-Guided RoI Extraction

Track-guided RoI extraction aligns the local features of the same trajectory across consecutive frames, providing a consistent spatial basis for temporal modeling. For candidate box $B_{i,k}$ of trajectory *i* in frame k∈{t−L+1,…,t}, RoIAlign extracts localized spatial features from pyramid level *s* [37]:

(13) $$F_{i,k}^{s}=\mathrm{RoIAlign}\left(P_{k}^{s},B_{i,k};H_{r},W_{r}\right)$$

where $P_{k}^{s}$ is the level-*s* feature map of frame *k*, and $H_{r}\times W_{r}$ defines the unified RoI spatial resolution, configured as 7×7 in this paper. Stacking these features across *N* trajectories and *L* frames yields the 5D tensor $F^{s}\in R^{N\times L\times C_{s}\times H_{r}\times W_{r}}$, where $C_{s}$ represents the channel dimension. These trajectory-aligned features provide a geometrically consistent foundation for capturing local structural evolution.

#### 3.3.2. Multi-Frame Temporal Feature Fusion

Temporal fusion is introduced to exploit the cross-frame evolution of target and deception responses, thereby reducing sensitivity to frame-wise fluctuations and providing additional cues for true–false discrimination. To model these temporal dependencies across consecutive frames, a multi-head self-attention (MHA) mechanism is employed [38]. At each pyramid level *s*, temporal modeling is performed independently at every spatial location within each RoI. Specifically, $F^{s}$ is first reshaped into sequence representations $X^{s}\in R^{\left(NH_{r}W_{r}\right)\times L\times C_{s}}$ by merging the trajectory and spatial dimensions. Furthermore, a learnable temporal positional encoding $E^{s}\in R^{1\times L\times C_{s}}$ is added to the sequence representation, forming the attention input $Z^{s}=X^{s}+E^{s}$. The respective query, key, and value matrices associated with the *h*-th head are derived through:

(14) $$Q_{h}^{s}=Z^{s}W_{h,s}^{Q},\;K_{h}^{s}=Z^{s}W_{h,s}^{K},\;V_{h}^{s}=Z^{s}W_{h,s}^{V}$$

The scaled dot-product attention is computed by:

(15) $$O_{h}^{s}=\mathrm{Softmax}\left(\frac{Q_{h}^{s}{\left(K_{h}^{s}\right)}^{⊤}}{\sqrt{d_{h}}}\right)V_{h}^{s}$$

where $d_{h}$ is the head dimension. The number of attention heads $H_{s}$ is configured according to the channel dimensionality of each pyramid level, specifically set to 4 for P3 and 8 for P4 and P5. The concatenated outputs are linearly projected:

(16) $$\mathrm{MHA}\left(Z^{s}\right)=\mathrm{Concat}\left(O_{1}^{s},\ldots ,O_{H_{s}}^{s}\right)W_{s}^{O}$$

To retain original frame features and stabilize optimization, a residual connection followed by layer normalization is applied:

(17) $${\tilde{X}}^{s}=\mathrm{LN}\left(Z^{s}+\mathrm{MHA}\left(Z^{s}\right)\right)$$

Subsequently, a mean aggregation across the *L* sequence steps compresses the temporal dimension:

(18) $${\bar{X}}^{s}=\frac{1}{L}\sum_{m=1}^{L}{\tilde{X}}_{:,m,:}^{s}$$

This operation outputs a refined temporal representation dynamically weighted by the self-attention mechanism, effectively mitigating single-frame anomalies. Finally, the aggregated sequence output is reshaped back into a 4D spatial tensor ${\bar{F}}^{s}\in R^{N\times C_{s}\times H_{r}\times W_{r}}$ to restore its structural layout.

#### 3.3.3. Cross-Scale Spatial Feature Fusion

Cross-scale spatial fusion combines fine localization details from shallow features with stronger semantic information from deeper features; relying on a single pyramid level would limit the balance between localization accuracy and discriminative semantics. Following temporal aggregation, the RoI features remain scale-specific. Although RoIAlign standardizes their spatial resolution to $H_{r}\times W_{r}$, features from different pyramid levels still differ in channel dimension, receptive field, and semantic content. Therefore, a scale-specific projection function $\phi_{s}\left(\cdot \right)$ maps each temporally aggregated feature into a common hidden dimension *D*:

(19) $$G^{s}=\phi_{s}\left({\bar{F}}^{s}\right)\in R^{N\times D\times H_{r}\times W_{r}}$$

Specifically, $\phi_{s}\left(\cdot \right)$ is implemented as a scale-specific 1×1 convolution followed by batch normalization and ReLU activation.

Because shallow features inherently preserve finer spatial localization and boundary details, the P3 level is designated as the reference scale ($s_{q}=3$). This reference is flattened to serve as the query *Q*, while the projected features from all pyramid levels constitute the multi-scale value set V:

(20) $$Q=\mathrm{Flatten}\left(G^{s_{q}}\right),\;V={\left\{G^{s}\right\}}_{s\in S}$$

To integrate cross-scale information, multi-scale deformable attention (MSDA) uses each query vector $z_{q}$ to predict the sampling offsets $\Delta p_{q,h,s,k}$ and attention weights $A_{q,h,s,k}$ through learnable linear projections, with the attention weights normalized by a softmax function. Therefore, for the *q*-th query vector $z_{q}$ at reference coordinate $p_{q}$, the aggregated output is:

(21) $$\mathrm{MSDA}\left(z_{q},p_{q},V\right)=\sum_{h=1}^{H_{ms}}W_{h}\left[\sum_{s\in S}\sum_{k=1}^{K}A_{q,h,s,k}\left(z_{q}\right)\;W_{h}^{\prime }B\left(G^{s},p_{q}^{s}+\Delta p_{q,h,s,k}\left(z_{q}\right)\right)\right]$$

where $H_{\mathrm{ms}}$ denotes the number of attention heads and *K* denotes the number of sampling points per pyramid level. $W_{h}^{\prime }$ and $W_{h}$ denote the head-specific value and output projection matrices, respectively. $p_{q}^{s}$ denotes the normalized reference position $p_{q}$ mapped to pyramid level *s*, and the bilinear interpolation operator B(·) samples feature values at fractional coordinates.

Finally, the output of MSDA applied over all queries *Q* and their corresponding normalized reference coordinates $P={\left\{p_{q}^{s}\right\}}_{q,s}$ is reshaped to the RoI spatial layout and combined with the reference feature $G^{s_{q}}$ via a residual connection and layer normalization:

(22) $$F_{\mathrm{STR}}=\mathrm{LN}\left(G^{s_{q}}+\mathrm{Reshape}\left(\mathrm{MSDA}\left(Q,P,V\right)\right)\right)$$

The final spatiotemporal trajectory representation $F_{\mathrm{STR}}\in R^{N\times D\times H_{r}\times W_{r}}$ combines shallow details with deep semantics for subsequent classification and bounding-box refinement.

#### 3.3.4. Classification and Regression Heads

The classification and regression heads translate the fused spatiotemporal representation $F_{\mathrm{STR}}$ into task-specific outputs for true–false discrimination and localization refinement. The classification head predicts one true target class, six deception classes, and an additional background class for invalid tracklet rejection. The regression head refines the last-frame candidate box by predicting non-negative normalized left, top, right, and bottom (LTRB) distances from its center. Assuming the reference box is parameterized by its center $\left(x_{i}^{a},y_{i}^{a}\right)$, width $w_{i}^{a}$, and height $h_{i}^{a}$, the corresponding regression mapping function is denoted by $g_{\mathrm{reg}}\left(\cdot \right)$. The predicted distance vector is given by $\delta_{i}=\left(\delta_{i}^{L},\delta_{i}^{T},\delta_{i}^{R},\delta_{i}^{B}\right)=\mathrm{Softplus}\left[g_{\mathrm{reg}}\left(F_{\mathrm{STR},i}\right)\right]$. The refined box is decoded as:

(23) $${\hat{B}}_{i}=\left(x_{i}^{a}-\delta_{i}^{L}w_{i}^{a},\;y_{i}^{a}-\delta_{i}^{T}h_{i}^{a},\;x_{i}^{a}+\delta_{i}^{R}w_{i}^{a},\;y_{i}^{a}+\delta_{i}^{B}h_{i}^{a}\right)$$

### 3.4. Adaptive R-D Parameter Extraction

In complex active deception-jamming environments, the local amplitude peak and the geometric center of a refined detection box provide two complementary priors for estimating target range and velocity. Peak-based extraction offers high localization accuracy when the energy of the target is well-focused, but the resulting estimate may be biased when a nearby strong deception response dominates the local amplitude distribution. Conversely, center-based extraction is robust to local amplitude anomalies, but its accuracy depends on bounding-box localization and the inverse mapping from image coordinates to the original R-D matrix. To exploit this complementarity, an adaptive peak-center fusion module is designed to learn dimension-specific fusion preferences from spatiotemporal features.

For a refined bounding box ${\hat{B}}_{i}$ in the R-D image, its geometric center is linearly mapped back to the discrete dimensions of the R-D matrix and subsequently converted to physical units to obtain the center prior $\left(R_{i}^{c},V_{i}^{c}\right)$. Meanwhile, the discrete indices (k,l) corresponding to the maximum amplitude within the bounded region in the original R-D amplitude matrix $A_{\mathrm{RD}}$ are extracted and converted to physical units, yielding the peak prior $\left(R_{i}^{p},V_{i}^{p}\right)$. The two priors are therefore represented in the same physical coordinate system and can subsequently be combined through adaptive fusion.

To dynamically fuse these two priors, global average pooling (GAP) is applied to compress the trajectory-level spatiotemporal feature $F_{\mathrm{STR},i}$ into a semantic vector $z_{i}$. A lightweight regression network $g_{\theta }\left(\cdot \right)$, followed by a sigmoid function σ(·), then maps this vector to dimension-specific fusion weights $\alpha_{R,i},\alpha_{V,i}\in \left(0,1\right)$:

(24) $$\left(\alpha_{R,i},\alpha_{V,i}\right)=\sigma \left(g_{\theta }\left(z_{i}\right)\right)$$

The final range and velocity estimates are obtained through adaptive fusion:

(25) $${\hat{R}}_{i}=\alpha_{R,i}R_{i}^{p}+\left(1-\alpha_{R,i}\right)R_{i}^{c}$$

(26) $${\hat{V}}_{i}=\alpha_{V,i}V_{i}^{p}+\left(1-\alpha_{V,i}\right)V_{i}^{c}$$

Thus, the contributions of the peak- and center-based estimates are adjusted independently for range and velocity according to the learned trajectory representation, reducing the dependence on either individual estimate.

### 3.5. Objective Functions and Training Strategy

#### 3.5.1. Objective Functions

STRNet is supervised by classification and bounding-box refinement. The objective is defined as:

(27) $$L_{\mathrm{II}}=L_{\mathrm{cls}}+\lambda_{L1}L_{L1}+\lambda_{\mathrm{CIoU}}L_{\mathrm{CIoU}}$$

where $L_{\mathrm{cls}}$ denotes the multi-class cross-entropy loss, $L_{L1}$ penalizes the normalized LTRB regression errors, and $L_{\mathrm{CIoU}}$ evaluates the overlap and geometric consistency. The coefficients $\lambda_{L1}$ and $\lambda_{\mathrm{CIoU}}$ balance the two box-refinement terms.

Furthermore, the adaptive fusion module predicts physical parameters exclusively for true targets. Let ${\hat{B}}_{i}$ and ${\hat{c}}_{i}$ denote the final refined bounding box and its predicted category output by Stage II, respectively, and let $k_{i}^{*}$ be the index of its assigned ground-truth annotation. To prevent deceptive responses and background false alarms from introducing invalid physical parameter supervision, the valid training set $S_{\mathrm{tar}}$ is defined as:

(28) $$S_{\mathrm{tar}}=\left\{i|{\hat{c}}_{i}=c_{\mathrm{tar}},c_{k_{i}^{*}}^{*}=c_{\mathrm{tar}}\right\}$$

where $c_{k_{i}^{*}}^{*}$ is the category of the matched ground-truth annotation, and $c_{\mathrm{tar}}$ denotes the true-target category.

For each valid target $i\in S_{\mathrm{tar}}$, the normalized measurement errors for range and velocity are evaluated as:

(29) $$e_{R,i}=\frac{{\hat{R}}_{i}-R_{k_{i}^{*}}^{*}}{\Delta R_{\mathrm{bin}}},\;e_{V,i}=\frac{{\hat{V}}_{i}-V_{k_{i}^{*}}^{*}}{\Delta V_{\mathrm{bin}}}$$

where ${\hat{R}}_{i}$ and ${\hat{V}}_{i}$ are the predicted physical parameters, $R_{k_{i}^{*}}^{*}$ and $V_{k_{i}^{*}}^{*}$ are the corresponding ground-truth values, and $\Delta R_{\mathrm{bin}}$ and $\Delta V_{\mathrm{bin}}$ denote the range and velocity resolutions. Let $N_{\mathrm{tar}}=\left|S_{\mathrm{tar}}\right|$ denote the total number of valid true-positive targets. The physical parameter regression loss is optimized using the Smooth L1 formulation:

(30) $$L_{\mathrm{param}}=\frac{1}{N_{\mathrm{tar}}}\sum_{i\in S_{\mathrm{tar}}}\left[\mathrm{SmoothL1}\left(e_{R,i}\right)+\mathrm{SmoothL1}\left(e_{V,i}\right)\right]$$

#### 3.5.2. Multi-Phase Optimization Strategy

To decouple the optimization of high-recall detection, fine-grained discrimination, and physical parameter estimation, the framework is trained through three sequential phases to minimize gradient interference:

- **Phase I: Candidate Detector Training.** The Stage I candidate detector is trained independently to extract frame-wise representations. Its weights are subsequently frozen to stabilize the input distribution for downstream temporal modules.
- **Phase II: STRNet Optimization.** With the detector frozen, STRNet is trained using the tracklets generated by the actual detection and association pipeline. This step exposes STRNet to realistic localization deviations and background noise, optimizing the classification and box-refinement objective ($L_{\mathrm{II}}$).
- **Phase III: Fusion Head Training.** Finally, both the detector and STRNet are frozen, and the adaptive fusion head is optimized via $L_{\mathrm{param}}$.

## 4. Experimental Results

### 4.1. Experimental Setup

#### 4.1.1. Dataset

Based on the signal generation mechanisms detailed in Section 2.1, a comprehensive multi-frame R-D domain simulation dataset was constructed. The dataset encompasses seven categories: a target-only scenario (NOJAM) and six scenarios involving typical active deception jamming techniques, including DFT, ISR, CI, SMSP, COMB, and RVGPO jamming. For all scenarios, the simulated raw echoes are processed through a unified radar signal processing chain. Following the preprocessing procedures detailed in Section 2.2, the resulting R-D matrices are converted into 640×640 grayscale image samples to serve as standard network inputs. The configuration parameters for the radar, target, and jamming models are summarized in Table 1. The dataset covers ${\mathrm{SNR}}_{\mathrm{RD}}$ values in {12,14,16,18,20}dB and JSR values in {0,4,8,12,16,20}dB. It contains 17,010 independent scene sequences, each comprising 10 temporally continuous R-D maps, yielding a total of 170,100 frames. The complete scene sequences are partitioned into the training, validation, and test sets at a 7:1:2 ratio, with all frames from each scene sequence assigned to the same subset. The 10-frame sequence length provides sufficient temporal observations for evaluating different temporal window lengths, and a five-frame window is used as the default model input in subsequent experiments.

#### 4.1.2. Metrics

The detection performance is evaluated using the probability of detection ($P_{d}$), false alarm probability ($P_{fa}$), and false deception ratio ($R_{fd}$), defined as:

(31) $$P_{d}=\frac{N_{\mathrm{TP}}}{N_{\mathrm{TP}}+N_{\mathrm{FN}}},\;P_{fa}=\frac{N_{\mathrm{FP}}}{N_{\mathrm{cell}}},\;R_{fd}=\frac{N_{D\to T}}{N_{T}}$$

where $N_{\mathrm{TP}}$, $N_{\mathrm{FN}}$, and $N_{\mathrm{FP}}$ represent the number of correctly detected true targets, missed true targets, and background false alarms, respectively; $N_{\mathrm{cell}}$ is the total number of evaluated R-D cells. $N_{D\to T}$ denotes the deceptive responses misclassified as true targets, and $N_{T}$ is the total number of true targets. $N_{\mathrm{FP}}$ and $N_{D\to T}$ represent mutually exclusive background false alarms and deceptive responses misclassified as true targets, respectively, while $R_{fd}$ is normalized by $N_{T}$ to measure the false-deception burden relative to the number of true targets.

To assess parameter estimation accuracy, the root mean square errors (RMSE) for range and velocity are calculated for the $N_{\mathrm{tar}}$ successfully matched true targets:

(32) $${\mathrm{RMSE}}_{R}=\sqrt{\frac{1}{N_{\mathrm{tar}}}\sum_{i=1}^{N_{\mathrm{tar}}}{\left({\hat{R}}_{i}-R_{i}\right)}^{2}},\;{\mathrm{RMSE}}_{V}=\sqrt{\frac{1}{N_{\mathrm{tar}}}\sum_{i=1}^{N_{\mathrm{tar}}}{\left({\hat{V}}_{i}-V_{i}\right)}^{2}}$$

where $\left(R_{i},V_{i}\right)$ and $\left({\hat{R}}_{i},{\hat{V}}_{i}\right)$ are the ground-truth and estimated range–velocity pairs of the *i*-th target, respectively.

Additionally, standard metrics including precision, recall, ${\mathrm{mAP}}_{50}$, and ${\mathrm{mAP}}_{50–95}$ are employed to comprehensively evaluate detection capabilities [39].

#### 4.1.3. Implementation Details

TSMF-Det is optimized following the three-phase training strategy described in Section 3.5.2. The training settings are summarized in Table 2. During evaluation, predictions with confidence scores above 0.70 were retained, and the matching IoU threshold was set to 0.50. All experiments were implemented in PyTorch (version 2.5.1) and executed on NVIDIA V100 GPUs. To ensure statistical reliability, all comparative and ablation experiments were repeated using five independent random seeds, and the reported results are presented as the mean ± standard deviation.

### 4.2. Detection Performance Analysis

**Figure 4:**
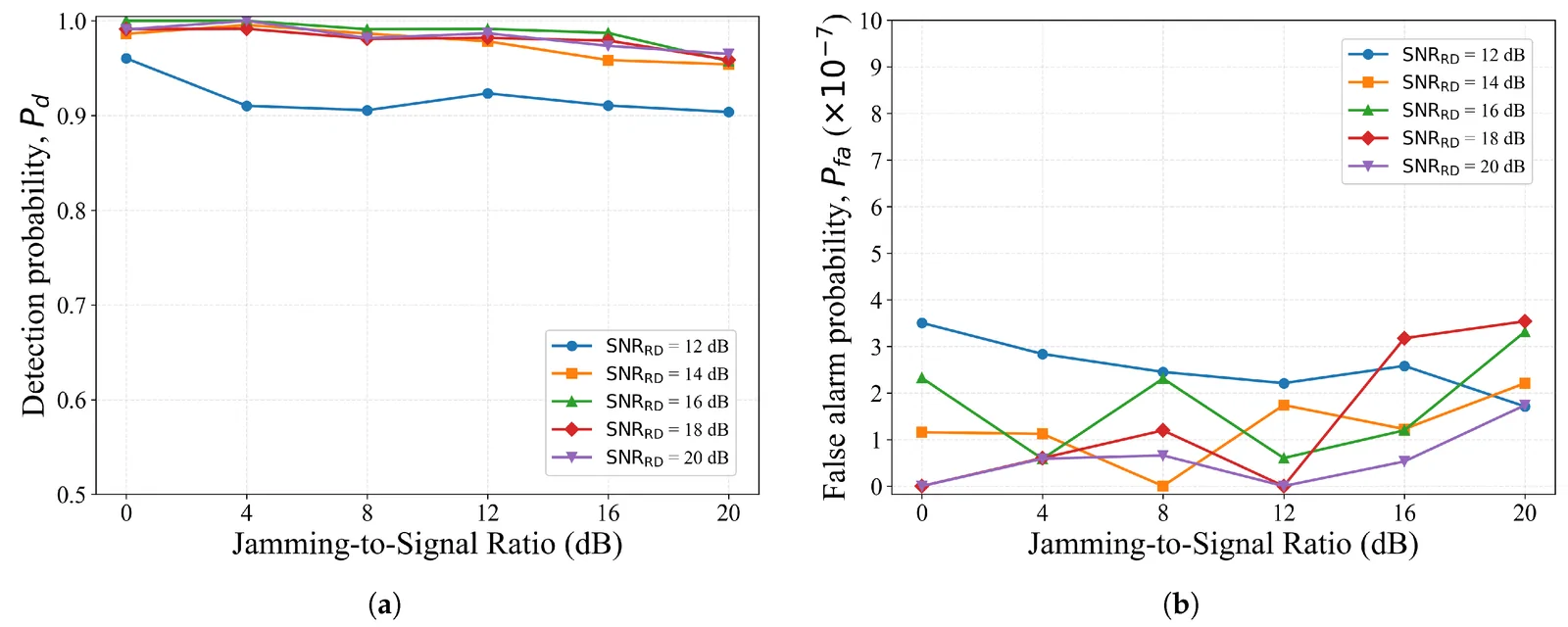

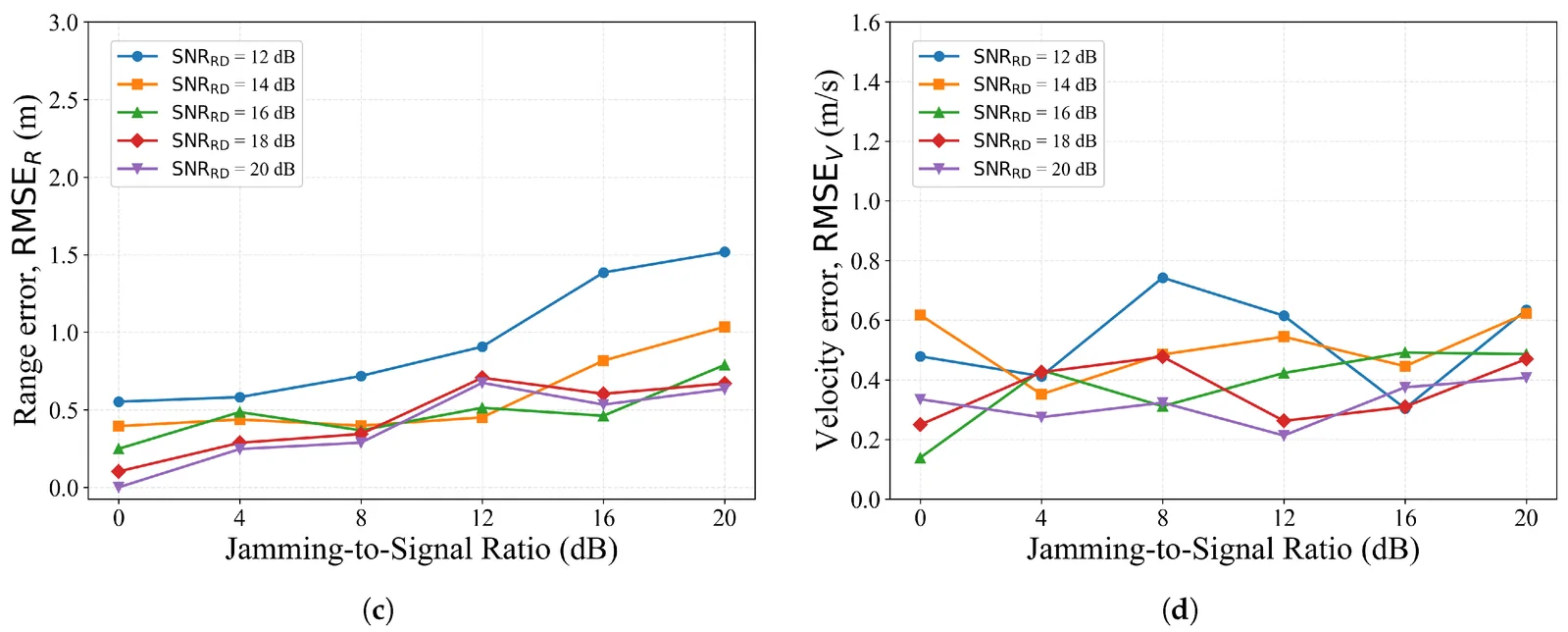
Detection performance and parameter extraction accuracy under varying ${\mathrm{SNR}}_{\mathrm{RD}}$ and JSR conditions. (**a**) detection probability, $P_{d}$; (**b**) false alarm probability, $P_{fa}$; (**c**) range error, ${\mathrm{RMSE}}_{R}$; (**d**) velocity error, ${\mathrm{RMSE}}_{V}$.

Figure 4 illustrates the detection probability, false alarm probability, and estimation errors for range and velocity across various levels of ${\mathrm{SNR}}_{\mathrm{RD}}$ and JSR. This evaluation investigates the robustness of TSMF-Det against the joint effects of weak target responses and severe deception jamming. As depicted in Figure 4a, when ${\mathrm{SNR}}_{\mathrm{RD}}\geq 14\;\mathrm{dB}$, $P_{d}$ remains consistently above 0.95. Even at ${\mathrm{SNR}}_{\mathrm{RD}}=12\;\mathrm{dB}$, where weak target responses become highly vulnerable to background noise and intense deceptive peaks, the detection probability is maintained above 0.90 across the entire JSR range. Furthermore, Figure 4b demonstrates that $P_{fa}$ remains on the order of $10^{-7}$, indicating that the high detection rate is achieved without substantially increasing background false alarms.

Figure 4c indicates that ${\mathrm{RMSE}}_{R}$ is sensitive to low ${\mathrm{SNR}}_{\mathrm{RD}}$ and high JSR conditions, especially when intense deceptive responses overlap with true targets in the range dimension, thereby amplifying errors during parameter extraction. Conversely, ${\mathrm{RMSE}}_{V}$ presented in Figure 4d exhibits fewer fluctuations and generally remains below 0.8m/s. Overall, while the confluence of low ${\mathrm{SNR}}_{\mathrm{RD}}$ and strong jamming exacerbates the difficulty of both target detection and parameter estimation, TSMF-Det sustains high $P_{d}$ and low $P_{fa}$ throughout the evaluated conditions.

Table 3 quantifies the performance of TSMF-Det across various deception jamming scenarios, confirming that distinct mechanisms impact detection and parameter extraction differently. Specifically, SMSP jamming induces large-scale spectral smearing that obscures true targets, yielding the lowest detection probability. Similarly, ISR jamming generates dense false responses near true targets, causing a lower detection probability, the highest false alarm probability, and increased range errors. In the remaining jamming scenarios, the detection probability reaches 0.99 while strictly maintaining a zero false deception ratio ($R_{fd}=0$). For RVGPO, the slightly higher velocity error aligns with its dynamic Doppler pull-off mechanism. Ultimately, TSMF-Det delivers superior performance against jamming with stable evolutionary patterns, as trajectory association and STRNet effectively exploit these spatiotemporal cues. Although performance inevitably drops slightly when targets are heavily obscured, the framework maintains competitive detection capabilities.

**Figure 5:**
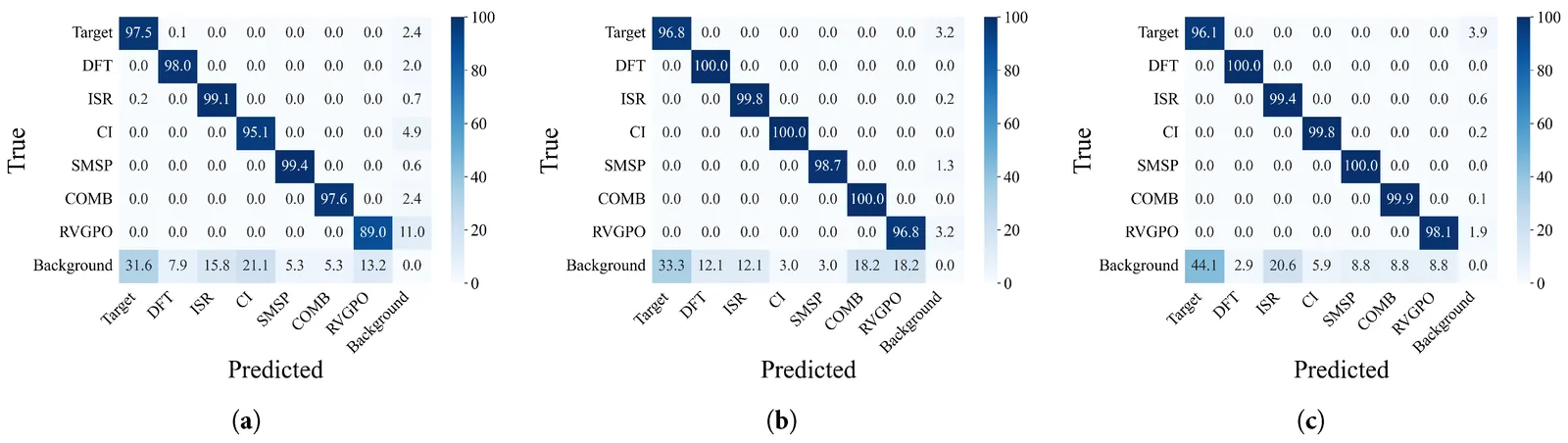
Normalized confusion matrices under different JSR conditions. (**a**) JSR = 0 dB; (**b**) JSR = 8 dB; and (**c**) JSR = 16 dB.

Figure 5 presents the normalized confusion matrices across varying JSR levels. True-target classification accuracy consistently exceeds 96%, exhibiting minimal degradation as jamming intensity increases. Inter-class confusion among the six deception categories remains negligible. Classification errors mainly appear as weak foreground responses being classified as background noise, instead of deceptive responses being misclassified as true targets. Specifically, weak deceptive responses at lower JSRs are occasionally misclassified as background, but their distinct temporal evolution becomes highly distinguishable as jamming power increases. Since the background class aggregates clutter and unmatched candidates, its distribution inherently differs from conventional foreground recall metrics.

### 4.3. Comparisons with Baseline Methods

Five representative methods are selected as baselines. SH-YOLOX-Nano [21] and LRRTDNet [23] are lightweight radar target detection methods, while YOLO11-N [40] and YOLO12-N [41] are included as recent general-purpose lightweight detectors. To provide a multi-frame comparison, YOLOv8-LSTM [26] is further included as a baseline that applies ConvLSTM-based temporal aggregation to YOLOv8 features under the same five-frame input setting.

Table 4 details the general object detection metrics. TSMF-Det demonstrates a substantial performance advantage over SH-YOLOX-Nano and LRRTDNet, whose lightweight and simplified architectures restrict their capacity to extract discriminative features in severe jamming environments. Among YOLO11-N, YOLO12-N, YOLOv8-LSTM, and TSMF-Det, the overall detection performance remains at a comparable level, while TSMF-Det shows a clearer advantage in recall. In particular, compared with YOLOv8-LSTM under the same five-frame input setting, TSMF-Det achieves better recall and ${\mathrm{mAP}}_{50–95}$, indicating that the performance gain is not attributable solely to the use of additional frames, but also to the trajectory-guided spatiotemporal refinement.

**Figure 6:**
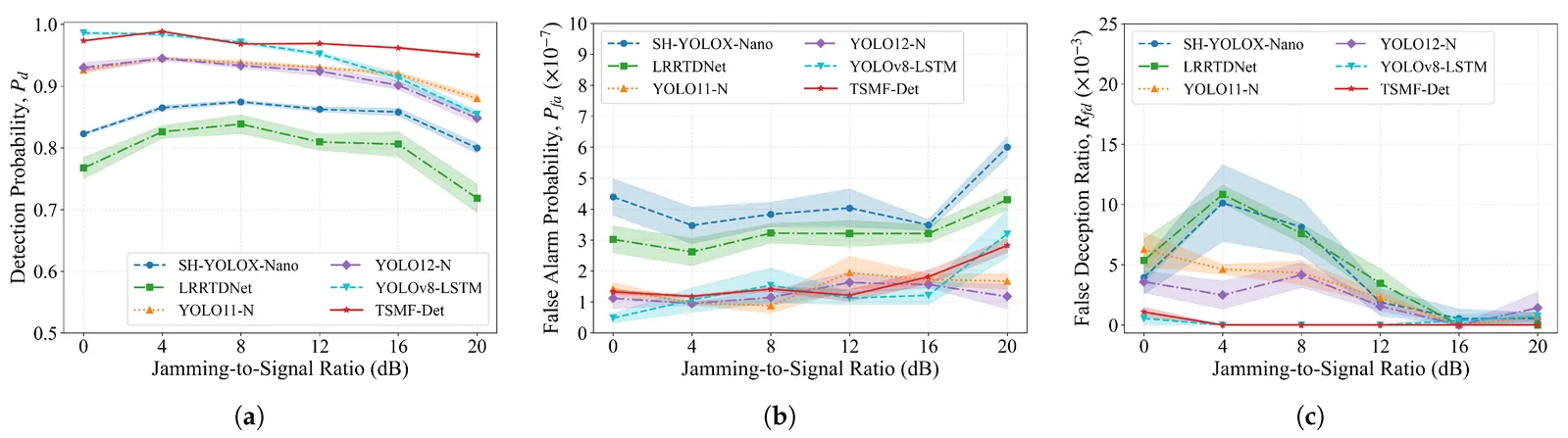
Detection performance of different methods under varying JSRs. (**a**) detection probability, $P_{d}$; (**b**) false alarm probability, $P_{fa}$; and (**c**) false deception ratio, $R_{fd}$.

Figure 6 compares the detection performance of different methods across various JSRs. As the JSR increases, the $P_{d}$ of the evaluated baseline methods generally decreases to varying degrees. TSMF-Det maintains a relatively stable $P_{d}$ across the evaluated JSR range, particularly under high JSR conditions. YOLOv8-LSTM achieves a high $P_{d}$ at low JSRs, but decreases progressively as the jamming intensity increases, with a more pronounced decline at high JSRs. In comparison, YOLO11-N and YOLO12-N exhibit lower $P_{d}$ and a sharper decline when the JSR reaches 20dB. Meanwhile, the radar-specific SH-YOLOX-Nano and LRRTDNet show lower overall $P_{d}$ and similarly degrade under high JSR conditions. For $P_{fa}$, the differences among TSMF-Det, YOLO11-N, YOLO12-N, and YOLOv8-LSTM remain relatively limited over most JSR conditions, whereas TSMF-Det maintains a consistently low $R_{fd}$. The comparison with YOLOv8-LSTM further suggests that ConvLSTM-based temporal aggregation alone does not fully prevent the degradation of $P_{d}$ under strong jamming.

**Figure 7:**
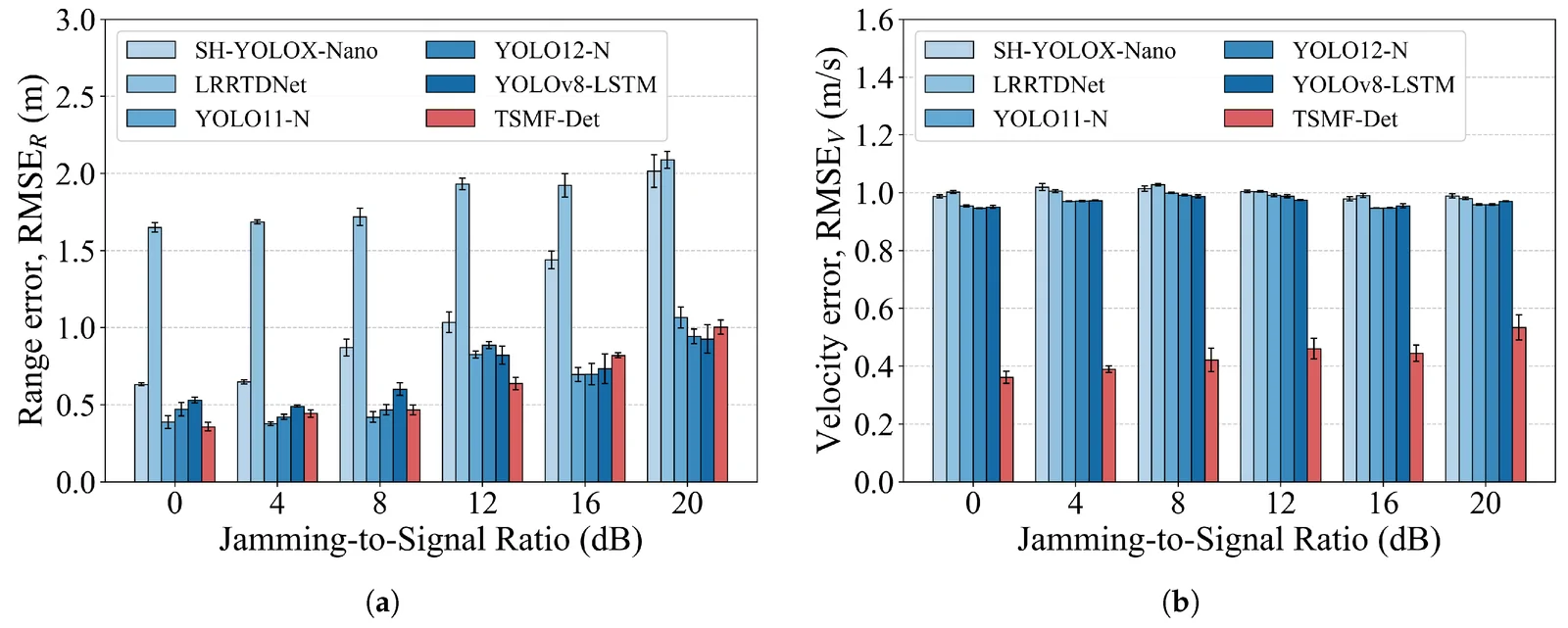
Physical parameter extraction errors of different methods under varying JSRs. (**a**) range error, ${\mathrm{RMSE}}_{R}$; (**b**) velocity error, ${\mathrm{RMSE}}_{V}$.

Figure 7 evaluates the range and velocity errors for all models. TSMF-Det maintains the lowest ${\mathrm{RMSE}}_{V}$ across the evaluated JSR conditions, while its ${\mathrm{RMSE}}_{R}$ remains generally lower than the radar-specific baselines and comparable to the recent YOLO-based methods. YOLOv8-LSTM, despite incorporating multi-frame temporal aggregation, exhibits parameter errors similar to those of the other detection baselines. This comparison suggests that temporal aggregation alone does not directly improve range–velocity estimation accuracy. The lower parameter errors achieved by TSMF-Det are consistent with its use of trajectory-level bounding-box refinement and adaptive parameter extraction, which reduces the dependence on geometric box centers for recovering R-D coordinates.

Table 5 compares the training and test performance of TSMF-Det with several strong baseline methods to assess the generalization gap. Overall, the train–test gaps of TSMF-Det remain comparable to those of YOLO11-N and YOLO12-N for both ${\mathrm{mAP}}_{50–95}$ and $P_{d}$, while being smaller than those of YOLOv8-LSTM. These results suggest that the additional trajectory association and spatiotemporal refinement do not lead to an evident increase in the generalization gap.

Table 6 assesses model complexity and inference efficiency. TSMF-Det requires 3.2 M parameters and 5.5 G FLOPs, representing a lower computational cost than the 6.5 G FLOPs required by YOLO11-N and YOLO12-N. Compared with the multi-frame YOLOv8-LSTM baseline, TSMF-Det substantially reduces both the parameters and FLOPs. Although SH-YOLOX-Nano and LRRTDNet require fewer parameters and shorter inference times, this lightweight design is achieved at the expense of detection robustness and parameter extraction accuracy. TSMF-Det is evaluated over the complete processing pipeline, including one current-frame candidate detection, five-frame trajectory association, STRNet inference, and adaptive parameter fusion, resulting in an inference time of 19.94 ms. Overall, this moderate temporal processing overhead is balanced by reliable true-target detection, deception rejection, and range–velocity estimation.

### 4.4. Ablation Study

#### 4.4.1. Contribution of Individual Components

Table 7 evaluates the contributions of trajectory association and the temporal and spatial fusion designs in STRNet. Introducing trajectory association improves $P_{d}$ and reduces $R_{fd}$, although retaining additional low-confidence candidates leads to a slight increase in $P_{fa}$. Replacing MHA with temporal convolution results in a larger performance reduction than replacing MSDA with direct concatenation, indicating that both temporal modeling and cross-scale spatial fusion contribute to the refinement process, with temporal fusion having a more pronounced effect under the current setting. The complete MHA–MSDA configuration provides the highest $P_{d}$, confirming the complementary roles of the two fusion components.

Table 8 compares four parameter extraction strategies using the same STRNet predictions. The fixed-fusion baseline uses $\alpha_{R}=\alpha_{V}=0.5$, corresponding to equal weighting of the center and peak estimates. Center extraction avoids dependence on local amplitude peaks but exhibits relatively large parameter errors, whereas peak extraction achieves lower velocity error at the cost of a substantial increase in range error. Fixed fusion provides only a limited compromise between the two estimates. In comparison, adaptive fusion achieves the lowest ${\mathrm{RMSE}}_{R}$ while maintaining a low ${\mathrm{RMSE}}_{V}$, demonstrating that dimension-specific weighting can better balance the complementary characteristics of center- and peak-based estimates.

#### 4.4.2. Impact of Temporal Window Length

Table 9 assesses the influence of temporal window length on model performance. As the number of fused frames increases, $P_{d}$ shows a gradual improvement, while $R_{fd}$ decreases markedly from three to five frames and remains at a low level thereafter. The gains beyond five frames are relatively limited, and $P_{fa}$ remains on the order of $10^{-7}$ across all evaluated window lengths. The physical parameter errors also vary only slightly across different window lengths, indicating that increasing the temporal window mainly benefits target detection and true–false discrimination, while range–velocity estimation is comparatively less sensitive to the number of fused frames.

## 5. Conclusions

To address the challenges of high missed detection rates, true–false target confusion, and inaccurate range–velocity estimation under mainlobe active deception jamming, a trajectory-guided two-stage multi-frame fusion detection (TSMF-Det) framework is proposed based on R-D images. Experiments covering six deception-jamming scenarios show that TSMF-Det maintains stable target detection and a low false deception ratio across varying JSR conditions, while achieving accurate range–velocity estimation. These performance improvements are primarily associated with the joint modeling of cross-frame continuity and multi-scale spatial features. Trajectory association establishes spatial correspondences among target responses across consecutive frames, while STRNet captures the distinct spatiotemporal evolution patterns of true targets and deceptive responses through temporal and spatial feature fusion. This design reduces the sensitivity of single-frame detection to energy fluctuations, noise masking, and strong deceptive responses. In addition, the adaptive parameter extraction module combines bounding-box centers with local amplitude peaks through dimension-specific fusion weights, balancing their complementary error characteristics for range and velocity estimation. The ablation results further show that temporal fusion and cross-scale spatial fusion both contribute to detection refinement, while adaptive peak-center fusion improves the balance between range and velocity estimation errors.

Nevertheless, multi-frame cascaded processing introduces additional computational cost and inference latency, and the current evaluation is primarily based on a simulated dataset. Future work will therefore investigate lightweight deployment and inference acceleration, together with validation on measured radar data, to improve real-time performance and generalization.

## Figures and Tables

**Table 1:** Simulation parameters for the radar system, target, and six types of active deception jamming.

	Parameters	Value
Radar & Target	Carrier frequency	16 GHz
	Pulse repetition interval	50 μs
	Pulse width	4–8 μs
	Pulse bandwidth	30–50 MHz
	Range-gate width	8–12 μs
	Target number	1–3
	Target velocity	±400 m/s
DFT	Number of false targets	4–10
	False target range interval	25–300 m
ISR	Slice cycle	0.5–2 μs
	Number of retransmissions	1–5
CI	Slice duration	1–3 μs
SMSP	Number of subpulses	2–5
COMB	Comb bandwidth	5–15 MHz
	Number of comb teeth	3–9
RVGPO	Range pull-off acceleration	20–30 m/s^2^
	Velocity pull-off acceleration	5–15 m/s^2^

**Table 2:** Implementation settings and hyperparameters of TSMF-Det.

Component	Parameter	Value
Candidate detector	Training epochs	100
	Batch size	128
	Optimizer	MuSGD
	Initial learning rate	$1\times 10^{-3}$
	Detection threshold $\tau_{\det}$	0.10
Tracker	High-confidence threshold	0.30
	Low-confidence threshold	0.10
	New-track threshold	0.20
	Matching threshold	0.80
STRNet	Training epochs	50
	Batch size	128
	Optimizer	AdamW
	Initial learning rate	$1\times 10^{-3}$
Adaptive fusion head	Training epochs	50
	Batch size	256
	Optimizer	AdamW
	Initial learning rate	$1\times 10^{-3}$

**Table 3:** Detection performance and parameter extraction accuracy across different jamming scenarios.

Metrics	NOJAM	DFT	ISR	CI	SMSP	COMB	RVGPO	Overall
$P_{d}$	0.94	0.99	0.96	0.99	0.93	0.99	0.99	0.97
$P_{fa}$ $\left(\times 10^{-7}\right)$	1.32	0.68	3.15	0.82	2.40	0.72	1.34	1.50
$R_{fd}$ $\left(\times 10^{-3}\right)$	0.00	0.00	1.04	0.00	0.00	0.00	0.00	0.15
${\mathrm{RMSE}}_{R}$ (m)	0.30	0.65	1.27	0.40	0.51	0.39	0.61	0.60
${\mathrm{RMSE}}_{V}$ (m/s)	0.39	0.43	0.37	0.44	0.39	0.49	0.55	0.44

**Table 4:** Comparison of detection performance with baseline methods.

Method	Precision (%)	Recall (%)	${\mathrm{mAP}}_{50}$ (%)	${\mathrm{mAP}}_{50–95}$ (%)
SH-YOLOX-Nano	96.57 ± 0.31	95.28 ± 0.08	97.29 ± 0.08	85.96 ± 0.19
LRRTDNet	95.96 ± 0.28	94.32 ± 0.37	96.85 ± 0.29	83.14 ± 0.81
YOLO11-N	98.03 ± 0.12	97.05 ± 0.07	98.61 ± 0.02	94.75 ± 0.04
YOLO12-N	98.02 ± 0.13	96.97 ± 0.26	98.52 ± 0.07	94.36 ± 0.23
YOLOv8-LSTM	98.00 ± 0.12	96.81 ± 0.24	98.64 ± 0.15	93.61 ± 0.17
TSMF-Det	98.68 ± 0.04	98.63 ± 0.02	98.30 ± 0.28	94.48 ± 0.16

**Table 5:** Comparison of training and test performance and generalization gaps across different methods.

Method	${\mathrm{mAP}}_{50–95}$ (%)			$P_{d}$ (%)		
	Train	Test	Gap	Train	Test	Gap
YOLO11-N	96.56 ± 0.09	94.75 ± 0.04	1.81 ± 0.10	94.91 ± 0.50	92.32 ± 0.24	2.59 ± 0.28
YOLO12-N	95.90 ± 0.20	94.36 ± 0.23	1.54 ± 0.06	92.98 ± 0.71	91.36 ± 0.48	1.63 ± 0.23
YOLOv8-LSTM	97.82 ± 0.22	93.61 ± 0.17	4.21 ± 0.21	97.97 ± 0.41	94.34 ± 0.29	3.63 ± 0.36
TSMF-Det	96.37 ± 0.08	94.48 ± 0.16	1.89 ± 0.04	98.92 ± 0.02	96.86 ± 0.03	2.06 ± 0.05

**Table 6:** Comparison of complexity and efficiency.

Method	Parameters (M)	FLOPs (G)	Time (ms)
SH-YOLOX-Nano	0.6	2.1	4.47
LRRTDNet	0.09	1.7	3.54
YOLO11-N	2.6	6.5	10.85
YOLO12-N	2.6	6.5	16.30
YOLOv8-LSTM	25.9	127.5	13.47
TSMF-Det	3.2	5.5	19.94

**Table 7:** Ablation of trajectory association and STRNet fusion components on detection performance. Upward and downward arrows indicate that higher and lower values are better, respectively. Checkmarks and crosses indicate whether the corresponding components are used or not, respectively.

Model Variant	Tracker	Temporal Fusion	Spatial Fusion	Detection Metrics		
				$P_{d}↑$ (%)	$P_{\mathrm{fa}}↓$ (×$10^{-7}$)	$R_{\mathrm{fd}}↓$ (×$10^{-3}$)
Candidate detector	×	×	×	92.12 ± 0.10	1.54 ± 0.03	4.40 ± 0.30
+Tracker	✓	×	×	94.04 ± 0.34	2.21 ± 0.18	3.99 ± 0.23
TConv variant	✓	TConv	MSDA	95.31 ± 0.08	1.57 ± 0.35	0.16 ± 0.13
Concat variant	✓	MHA	Concat	96.73 ± 0.06	1.53 ± 0.05	0.15 ± 0.06
Full STRNet	✓	MHA	MSDA	96.86 ± 0.03	1.63 ± 0.07	0.18 ± 0.07

**Table 8:** Ablation of physical parameter extraction strategies. Downward arrows indicate that lower values are better.

Parameter Extraction Strategy	${\mathrm{RMSE}}_{R}↓$ (m)	${\mathrm{RMSE}}_{V}↓$ (m/s)
Center extraction	0.87 ± 0.01	0.97 ± 0.01
Peak extraction	1.76 ± 0.05	0.40 ± 0.01
Fixed fusion	1.03 ± 0.02	0.52 ± 0.00
Adaptive fusion	0.68 ± 0.04	0.43 ± 0.01

**Table 9:** Effect of temporal window length on detection performance and parameter extraction accuracy.

Frames	Detection Metrics			Accuracy Metrics	
	$P_{d}\;\left(\%\right)$	$P_{\mathrm{fa}}\;(\times$ $10^{-7})$	$R_{\mathrm{fd}}\;(\times$ $10^{-3})$	${\mathrm{RMSE}}_{R}$ (m)	${\mathrm{RMSE}}_{V}$ (m/s)
3	96.23 ± 0.09	1.43 ± 0.06	0.62 ± 0.12	0.63 ± 0.01	0.44 ± 0.00
5	96.86 ± 0.03	1.63 ± 0.07	0.18 ± 0.07	0.68 ± 0.04	0.43 ± 0.01
7	97.05 ± 0.03	1.70 ± 0.12	0.18 ± 0.12	0.64 ± 0.01	0.45 ± 0.00
9	97.71 ± 0.17	1.57 ± 0.19	0.23 ± 0.14	0.62 ± 0.00	0.46 ± 0.00

## Data Availability

The data are not publicly available due to the fact that they currently include privileged information.
